# 

**Published:** 1889-12

**Authors:** 


### BERKSHIRE.

MAIDENHEAD.
J. Lidderdale

### CHESHIRE.

CHESTER.

R. E. Burges

EGREMONT.

T. W. A. Napier

### CORNWALL.

CALSTOCK.

H. T. Woodd

CAMBORNE.
J. Paull

HELSTON.
J. W. Norman

LOSTWITHIEL.
J. H. Sewart

NEW QUAY.
A. Hardwick

PADSTOW.
H. F. Marley

PENZANCE.
W. S. Bennett

POLRUAN.
W. H. Torbock

PORT ISAAC.
C. Williams.

REDRUTH.
T. B. Carlyon
J. S. Hichens
G. A. Michell

ST. AUSTELL.

W. Mason

ST. JUST.
R. B. Searle

TRURO.

J. B. Jackson

E. Rundle

### DEVONSHIRE.

BAMPTON.
T. A. Guinness

BARNSTAPLE.
C. H. Gamble
J. Harper
W. A. G. Laing

BIDEFORD.

W. H. Ackland

BRIXHAM.
G. C. Searle

BROADCLYST.
J. Somer

CHAGFORD.
A. D. Hunt

DAWLISH.

A. de W. Baker

F. M. Cann

DEVONPORT.
G. T. Rolston
F. E. Row

EXBRIDGE.
G. F. Sydenham

EXETER.
J. M. Ackland
A. G. Blomfield
P. M. Deas
A. W. Kempe
L. Shapter
E. B. Steele-Perkins
L. H. Tosswill

HATHERLEIGH.
H. H. Parsloe

HONITON.
T. W. Shortridge

HORRABRIDGE.
A. P. Prowse

LYNTON.
F. C. Berry

NEWTON ABBOT.
E. Haydon
W. G. Scott

OTTERY ST. MARY.
F. A. Gray

PAIGNTON.
J. Alexander

### DEVONSHIRE (*Continued*).

PLYMOUTH.
R. H. Clay
E. E. Meeres
W. G. Nash
E. B. Thomson

PLYMPTON.
W. D. Stamp

ST. MARY CHURCH.
T. Finch

SALCOMBE.
A. H. Twining

SOUTH MOLTON.
E. Furse

TAVISTOCK.
M. T. Leamon

TIVERTON.
J. S. Smith

TORQUAY
W. Powell

TOTNES.
D. A. Fraser

UFFCULME.
F. Morgan

WINKLEIGH.
J. H. Norman

### DORSETSHIRE.

BEAMINSTER.
T. P. Daniel

BOURTON.
B. P. Bartlett

CERNE ABBAS.
E. W. Kerr

DORCHESTER.
F. B. Fisher
P. W. MacDonald

POOLE.
J. McNicoll

PORTLAND.
G. H. Lilley

SHERBORNE.
W. H. Williams

STURMINSTER NEWTON.
J. C. Leach

WEYMOUTH.
R. W. Carter
J. M. Lawrie
W. G. V. Lush

WIMBORNE.
A. J. H. Crespi

### ESSEX.

SOUTHEND.
E. E. Phillips

### GLOUCESTERSHIRE.

BRISTOL.

W. R. Ackland
G. Adams
J. B. Adams
G. F. Atchley
W. M. Barclay
T. G. L. Baretti
B. J. Baron
J. Beddoe
A. E. Blacker
A. F. Blagg
E. C. Board
J. Broom
G. F. Burder
J. P. Bush
F. Calder
J. Cameron
A. Carr
W. F. Carter
J. M. Clarke
E. W. Coathupe
R. W. Coe
T. V. Coker
T. J. Colman
G. G. Corbould
W. Cotton
F. R. Cross
J. Dacre
D. S. Davies
H. R. Dew
H. F. Devis
N. C. Dobson
W. Dowson
E. L. W. Dunbar
W. Duncan
T. F. Edgeworth
W. Elder
C. Elliott
W. R. Etches
J. Ewens
R. G. Fendick
E. L. Fox
W. J. Fyffe
G. Gardiner
A. N. G. Gibbs
J. Gill
G. A. Gloag
W. C. Goodden
W. G. Grace
L. M. Griffiths
C. D. G. Hailes
A. J. Harrison
W. H. Harsant
A. G. Hayman
J. C. Heaven
C. K. C. Herapath
H. E. Hetling
H. Hill
F. F. Hills
General Hospital
G. A. Imlay
Royal Infirmary
F. P. Lansdown
A. E. A. Lawrence
H. Lawrence
E. L. Lees
Museum and Library
W. E. Lloyd
F. T. B. Logan
W. T. Maddison
H. Marshall
W. H. Masters
C. E. Matthews
T. O. Mayor
J. S. Metford
J. Milne
C. A. Morton
W. N. Nevill
C. Newman
W. H. C. Newnham
J. Newstead
T. D. Nicholson
J. A. Norton
G. S. Page
G. Parker
J. H. Parry
T. C. Parson
G. C. P. Pauli
F. Penny
J. H. Perkins
C. F. Pickering
W. E. Pountney
A. Prichard
A. W. Prichard
J. E. Prichard
A. Pring
A. B. Prowse

### GLOUCESTERSHIRE *(Continued).*

BRISTOL *(continued).*

W. B. Roué
C. K. Rudge
H. T. Rudge
H. F. Semple
J. E. Shaw
E. J. Sheppard
W. E. Shorland
E. M. Skerritt
G. M. Smith
J. G. Smith
R. S. Smith
S. Smith
G. M. Stansfeld
C. Steele
F. W. Stoddart
J. Swain
J. G. Swayne
S. H. Swayne
W. C. Swayne
J. Taylor
R. W. Thomas
W. J. Tivy
H. H. Tomkins
H. Waldo
C. H. Wallace
J. H. Wathen
T. Webster
C. E. Wedmore
R. R. Whishaw
J. Wilding
P. W. Williams
H. W. Windsor-Aubrey

CHALFORD.

F. C. Palmer

CHELTENHAM.

J. W. Bramwell
J. C. Gooding
Cheltenham Library
C. J. Newton
F. A. A. Smith
L. Winterbotham

CHIPPING SODBURY.

A. Grace

CINDERFORD.

W. C. Heane

COLEFORD.

L. B. Trotter

DOWNEND.

H. Skelton

FISHPONDS.

W. Brown

GLOUCESTER.

R. W. Batten
E. D. Bower
T. S. Ellis
E. I. Rowe

HAMBROOK.

E. Crossman
W. F. B. Eadon

HAWKESBURY-UPTON.

W. I. Cox

KINGSWOOD.

H. Grace

LYDBROOK.

H. S. Fletcher

LYDNEY.

A. S. Currie

MORETON-IN-MARSH.

L. K. Yelf

STAPLE HILL.

W. Murray

STAPLETON.

H. A. Benham
G. Thompson

STOKE BISHOP.

J. Stewart

STONEHOUSE.

G. T. B.Watters

STOW-ON-THE-WOLD.

E. Dening

STROUD.

A. S. Cooke
E. A. Howsin
W. H. Paine
F. W. Storry

TETBURY.

W. Wickham

THORNBURY.

E. M. Grace
T. H. Taylor

WESTBURY-ON-TRYM.

H. Ormerod
A. W. Riddell

WINTERBOURNE.

R. Eager

WOTTON-UNDER-EDGE.

D. H. Forty

### HAMPSHIRE.

BEMBRIDGE.

T. D. Pain

BOURNEMOUTH.

D. C. Embleton
S. T. Smyth

EMSWORTH.

L. Stephens

LYMINGTON.

J. Rendall

SOUTHAMPTON.

Army Medical Staff Library

SOUTHSEA.

J. W. Cousins

VENTNOR.

R. Robertson

### HEREFORDSHIRE.

HEREFORD.

W. C. Whitfeld

### HERTFORDSHIRE.

ST. ALBANS.

A. H. Boys

### KENT.

TUNBRIDGE WELLS.

C Wilson.

### LANCASHIRE.

LIVERPOOL.
Medical Institution

POULTON-LE-FYLDE.
P. H. Day

### MIDDLESEX.

ISLEWORTH.
W. A. Moynan.

LONDON.
J. Abercombie
L. S. Beale
L. Browne
F. Cock
H. Cooper
J. F. Evans
A. P. Gould
G. S. Gregory
M. Grigor
G. Hett
C. M. Jessop
J. Lister
W. C.Luffman
W. Pippette
W. J. Penny
M. W. H. Russell
W. J. Seward
Royal Med. and Chir. Society
G. D. Thomas
J. A. Watson

TOTTENHAM.
W. H. Plaister

### MONMOUTHSHIRE.

ABERGAVENNY.
S. H. Steel

EBBW VALE.
J. W. Davies

NANTYGLO.
T. W. Bevan

NEWPORT.
W. Basset
H. R. Hudson
T. Limbery
O. E. B. Marsh
A. G. Thomas
H. E. Williams

PONTNEWYDD.
R. Edmunds

PONTYPOOL.
S. B. Mason
E. S. Wood

RHYMNEY.
T. H. Redwood

RISCA.
G. B. Robathan

TREDEGAR.
G . A. Brown

### OXFORDSHIRE.

OXFORD.
R. W. Doyne

### SOMERSETSHIRE.

BANWELL.
A. J. Bisdée

BATH.
H. T. S. Aveline
W. B. Beatson
A. B. Brabazon
C. Coates
T. Cole
F. S. Cowan
S. Craddock
J. Davies
R. Davis
W. M. Ellis
A. E. W. Fox
H. W. Freeman
H. F. A. Goodridge
T. B. Goss
F. K. Green
F. P. Hoblyn
H. C. Hopkins
L. J. King
F. Lace
T. P. Lowe
A. L. Marshall
R. J. H. Scott
E. Skeate
J. K. Spender
L. Weatherly

BECKINGTON.
W. G. Evans

BRENT KNOLL.
J. W. Papillon

BRIDGWATER.
F. J. C. Parsons
J. B. Sincock

BRISLINGTON.
B. B. Fox
C. H. Fox

BRUTON.
F. Stockwell

BURNHAM.
A. W. C. Peskett

CHEDDAR.
R. W. Statham

CHEW MAGNA.
E. T. Hale

CLEVEDON.
T. Davis
G. F. P. Pizey
S. Skinner

CREWKERNE.
E. J. Cave
W. W. Webber

### SOMERSETSHIRE *(Continued)*.

DULVERTON.
R. J. Collyns

FRESHFORD.
C. E. S. Flemming.

FROME.
F. Parsons

GLASTONBURY.
J. A. Bright

ILMINSTER.
C. Munden

KEYNSHAM.
J. Lodge
G. G. D. Willett

LANGPORT.
J. Morgan

MIDSOMER NORTON.
E. Blacker
A. Waugh

MILVERTON.
C. Randolph

MINEHEAD.
T. Clark
T. J. Ollerhead

NORTH PETHERTON.
C. F. Hawkins

PILL.
R. A. Ross

PORTISHEAD.
W. Monckton
C. A. Wigan

SHEPTON MALLET.
B. N. Hyatt

SOUTH PETHERTON.
W. H. Walter

STOGUMBER.
C. E. S. Brettingham

STREET.
A. Clarke

TAUNTON.
H. J. Alford
J. Carey
Taunton Hospital
W. Liddon

TEMPLE CLOUD.
T. Martin

TWERTON-ON-AVON.
J. Wigmore

WEDMORE.
J. Ford
R. P. Tyley

WELLINGTON.
J. Meredith
T. E. P. Prideaux

WELLS.
W. Fairbanks
A. L. Wade

WESTON-SUPER-MARE.
G. E. Alford
E. F. H. Burroughs
G. B. Fraser
E. F. Martin
W. H. G. Phelps
G. F. Rossiter
R. Roxburgh
F. W. S. Wicksteed

WRINGTON.
A. H. Benson
H. W. Collins

YATTON.
A. de C. Lyons

YEOVIL.
P. S. H. Colmer
C. J. Marsh
E. Vernon

### STAFFORDSHIRE.

LONGTON.
E. D. Duffett

STAFFORD.
J. Scott

### SURREY.

ESHER.
J. B. Fry

SURBITON.
J. G. Davey

### SUSSEX.

ST. LEONARD'S-ON-SEA.
H. A. Spencer
W. H. Spencer

### WARWICKSHIRE.

BIRMINGHAM.
L. Tait

### WILTSHIRE.

BURBAGE.

A. E. N. Browne

CALNE.
W. S. Batten

DEVIZES.

T. B. Anstie

C. Eddowes

A. H. Syrée

FOVANT.
C. Clay

MALMESBURY.
R. Kinneir

SALISBURY.
H. Coates
L. S. Luckham
H. J. Manning

STRATTON ST. MARGARET.
J. Fernie

SWINDON.
W. Howse
J. C. Maclean

WARMINSTER.
J. Hinton
R. L. Willcox

WOOTTON BASSETT.
J. M. Kirkman

### WORCESTERSHIRE.

WORCESTER.
J. F. Royle

### YORKSHIRE.

WAKEFIELD.
A. R. Steel

YORK.
L. H. Williams

### Ireland.

### ANTRIM.

BELFAST.
J. W. Browne

### LOUTH

LOUTH.
J. Browne

### Scotland.

### EDINBURGHSHIRE.

EDINBURGH.
Y. J. Pentland

### Wales.

### BRECONSHIRE.

BRECON.
A. Whyte

BRYNMAWR.
G. H. Browne

SENNY BRIDGE.
W. R. Jones

TALGARTH.
W. Howells

### CARDIGANSHIRE.

LAMPETER.
E. C. Davies

NEW QUAY.
E. R. Jones

### CARMARTHENSHIRE.

CARMARTHEN.
A. Devonald
G. J. Hearder

FERRYSIDE.
P. Williams

LLANELLY.
D. J. Williams

### GLAMORGANSHIRE.

ABERAMAN.
J. B. James

ABERAVON.
J. A. Jones

ABERDARE.
E. Jones

CAERPHILLY.
J. Lewellyn

CARDIFF.
W. T. Edwards
G. W. Grote
J. Milward
W. Morgan
D. R. Paterson
W. Price
R. Prichard
A. Sheen
Medical Society
J. T. Thompson
C. T. Vachell
H. R. Vachell

LLANDAFF.
J. Arthur

MERTHYR TYDVIL.
J. L. W. Ward

PENARTH.
W. J. Clapp

PORTH.
H. N. Davies.

REYNOLDSTONE.
H. V. Ellis

SWANSEA.
G. Padley
J. A. Rawlings
J. Thomas

### MONTGOMERYSHIRE.

LLANSAINTFFRAID.
T. Edwardes

### PEMBROKESHIRE.

TENBY.
B. Kendall

### Channel Islands.

GUERNSEY.
N. Webster.

### Italy.

PALERMO.
C. Scimonelli

### Switzerland.

DAVOS-PLATZ.
W. R. Huggard

### INDEX.

Accidental hæmorrhage—Dr. J. G. Swayne, 170.
Acne, Treatment of (*Extract*), 139.
Acromegalie (*Extract*), 137.
Alexander, Dr. W.—Treatment of epilepsy (*Review*), 271.
Ambulance work—Dr. R. L. Roberts (*Review*), 50.
Anæsthetics in naso-pharyngeal operations (*Extract*), 284.
Annual of the universal medical sciences (*Review*), 268.
Annual, The Medical (*Review*), 55.
Antipyrin (*Extract*), 65.
Army-surgeon, Reminiscences of an —Dr. W. J. Fyffe, 145.

Baron, Dr. B. J.—Treatment of laryngeal growths, 78.
Bath and her thermal waters—S. Craddock (*Review*), 273.
Beddoe, Dr. J.—Clifton, 114.
Berry, Dr. G. A.—Diseases of eye (*Review*), 207.
Bigelow, Dr. H. R.—Gynæcological electro-therapeutics (*Review*), 269.
Booksellers, Directory of second-hand (*Review*), 214.
Bournemouth—A. J. H. Crespi, 36; A. Kinsey-Morgan (*Review*), 124.
Bourneville and Bricon, Drs.—Manual of hypodermic medication (*Review*), 46.
Bright's disease—Dr. R. Saundby (*Review*), 129.
Bryant, T.—Lectures on tension, &c. (*Review*), 50.

Caird and Cathcart, Drs.—Surgical handbook (*Review*), 267.
Calomel (*Extract*), 70.
Campbell, Dr. H.—Causation of disease (*Review*), 53.
Cancer and its complications—C. E. Jennings (*Review*), 274.
Cancer of dura mater (*Extract*), 136.
Cancer, Tissue-metabolism and coma in (*Extract*), 281.
Carcinoma, Surgical aspects of—N. C. Dobson, 225.
Causation of disease — Dr. H. Campbell (*Review*), 53.
Chloral, Hydrate of—Dr. J. K. Spender, 1.
Clarke, Dr. J. M.—Congenital heart-disease, 261; Dilatation of stomach, 21; Disseminated sclerosis, 265; Myxœdema, 258; Tubercle of medulla oblongata, 194.
Clifton—Dr. J. Beddoe, 114.
Cocaine poisoning (*Extract*), 136.
Craddock, S.—Bath and her thermal waters (*Review*), 273.
Crespi, A. J. H.—Bournemouth, 36; Inebriety, 244.

Dale, Dr. R.—Epitome of surgery (*Review*), 49.
Diabetes—Dr. E. Schnée (*Review*), 211.
Diary, Medical (*Review*), 271.
Diet, The normal—G. M. Smith, 73.
Diphtheria, Infection in (*Extract*), 217.

Disseminated sclerosis—Dr. J. M. Clarke, 265.
Dobson, N. C.—Cases of oral surgery, 108; Surgical aspects of carcinoma, 225.
Domestic animals, Physiology of—Dr. R. M. Smith (*Review*), 267.
Dowse, Dr. T. S.—Massage and electricity (*Review*), 127.

Ear, Acute inflammation of middle—C. F. Pickering, 32.
Ellis, T. S.—The human foot (*Review*), 268.
Epilepsy, Treatment of—Dr. W. Alexander (*Review*), 271.
Exophthalmic goitre (*Extract*), 222.
Eye, Diseases of—Dr. G. A. Berry (*Review*) 207.

Fever—Dr. T. J. Maclagan (*Review*), 48.
Flint, Dr. A.—Textbook of physiology (*Review*), 127.
Foot, The human—T. S. Ellis (*Review*), 268.
Fyffe, Dr. W. J.—Reminiscences of an army-surgeon, 145.

Gout—Dr. R. Roose (*Review*), 270.
Gynæcological electro-therapeutics—Dr. H. R. Bigelow (*Review*), 269.

Heart-disease, Case of congenital—Dr. J. M. Clarke, 261
Hernia, Lectures on—C. B. Lockwood (*Review*), 272.
Higgens, C.—Ophthalmic practice (*Review*), 55.
Histology, Elements of-Dr. E. Klein (*Review*), 126.
Hitchins, C. V.—Weston-super-Mare, 198.
Höhenklima, Das—Dr. A. Ladendorf (*Review*), 130.
Hydracetin (*Extract*), 140.
Hygiene and public health—Dr. L. C. Parkes (*Review*), 272.
Hypnotic sleep, Removal of vaginal cystocele during (*Extract*), 282.
Hypnotics, New (*Extract*), 283.
Hypodermic medication, Manual of—Drs. Bourneville and Bricon (*Review*), 46.

Ileus, Treatment of (*Extract*), 220.
Inebriety—A. J. H. Crespi, 244.
Instruments in operations—A. M. Robson (*Review*), 124.
International medical congress in 1890, 214.
Intestinal diseases of infancy and childhood—Dr. A. Jacobi (*Review*), 47.
Iodol (*Extract*), 64.

Jacobi, Dr. A.—Intestinal diseases of infancy and childhood (*Review*), 47.
Jacobson, W. H. A.—The operations of surgery (*Review*), 49.
James, Dr. P.—Alterations in the British Pharmacopœia (*Review*), 270.
Jennings, C. E.—Cancer and its complications (*Review*), 274.
Jollye, F. W.—Birth palsy, 87.

Kinsey-Morgan, A.—Bournemouth (*Review*), 124.
Klein, Dr. E.—Elements of histology (*Review*), 126.

Laboratory reports, Edinburgh (*Review*), 126.
Ladendorf, Dr. A.—Das höhenklima (*Review*), 130.
Laryngeal growths, Treatment of—Dr. B. J. Baron, 78.
Lewers, Dr. A. H. N.—Diseases of women (*Review*), 51.
Lockwood, C. B.—Lectures on hernia (*Review*), 272.

Maclagan, Dr. T. J.—Fever (*Review*) 48.
Maddox, Dr. E. E.—Clinical use of prisms (*Review*), 209.
Massage—Dr. H. Tibbits (*Review*), 54.
Massage and Electricity—Dr. T. S. Dowse (*Review*), 127.
Masso-therapeutics—Dr. W. Murrell (*Review*), 127.
McVail, Dr. J. C.—Vaccination vindicated (*Review*), 44.
Medicine, Practical—Dr. A. H Carter (*Review*), 123.

Medicine, Textbook of — Dr. A. Money (*Review*), 123.
Medulla oblongata, Tubercle of— Dr. J. M. Clarke, 194.
Murrell, Dr. W.—Masso-therapeutics (*Review*), 127; What to do in cases of poisoning (*Review*), 271
Museum for British Medical Association visit in 1891, 142.
Myrtol (*Extract*), 63.
Myxœdema, Case of — Dr. J. M. Clarke, 258.

Naso-pharyngeal operations, Anæsthetics in (*Extract*), 284.

Obesity (*Extract*), 218.
Ophthalmic practice—C. Higgens (*Review*), 55.
Oral surgery, Cases of — N. C. Dobson, 108.
Osteo-arthritis—Dr. J. K. Spender (*Review*), 212.

Palsy, Birth—F. W. Jollye, 87.
Parkes, Dr. L. C.—Hygiene and public health (*Review*), 272.
Pharmacopœia, Alterations in the British — Dr. P. James (*Review*), 270.
Pharyngitis, Chronic—Dr. P. W. Williams, 189.
Phenacetin (*Extract*), 67.
Phthisis, Functions of stomach in, 219.
Physiology, Text-book of—Dr. A. Flint (*Review*), 127.
Pickering, C. F.— Acute inflammation of middle ear, 32.
Poisoning, What to do in cases of— Dr. W. Murrell (*Review*), 271.
Pregnancy, Ectopic—L. Tait (*Review*), 50.
Preparations for the Sick, Notes on —56, 131, 215, 276.
Prisms, Clinical use of—Dr. E. E. Maddox (*Review*), 209.
Progressive muscular atrophy (*Extract*), 134.
Psycho-therapeutics — Dr. C. L. Tuckey (*Review*), 269.
Purdon, Dr. H. S.—Diseases of skin (*Review*), 213.

Remedies, Recently introduced (*Review*), 214.
Ringer, Dr. S.—Handbook of therapeutics (*Review*), 210.
Roberts, Dr. R. L.— Ambulance work (*Review*), 50.
Robson, A. M. — Instruments in operations (*Review*), 125.
Roose, Dr. R.—Gout (*Review*), 270.
Rumination (*Extract*), 61.

Saundby, Dr. R.— Bright's disease (*Review*), 129.
Schnée, Dr. E.—Diabetes (*Review*), 211.
Schultze, Dr. B. S.— Displacements of uterus (*Review*), 52.
Scraps, 71, 143, 223, 287.
Skene, Dr. A. J. C.—Diseases of women (*Review*), 125.
Skin, Diseases of—Dr. H. S. Purdon, (*Review*) 213.
Smith, Dr. R. M.— Physiology of domestic animals (*Review*), 267.
Smith, G. M.—The normal diet, 73.
Sozoiodol (*Extract*), 65.
Spender, Dr. J. K.—Hydrate of chloral, 1; Osteo-arthritis (*Review*), 212.
Stomach, Dilatation of—Dr. J. M. Clarke, 21; washing out (*Extract*), 66.
Strophanthus (*Extract*), 69.
Sulphonal (*Extract*), 221.
Surgery, The operations of—W. H. A. Jacobson (*Review*), 49; Epitome of—Dr. R. Dale (*Review*), 49.
Surgical handbook — Drs. F. M. Caird and C.W. Cathcart (*Review*), 267.
Swayne, Dr. J. G.—Accidental hæmorrhage, 170.
Syphilis treated hypodermically (*Extract*), 141.
Syphilis, Iodide of potassium in — (*Extract*), 282.

Tabes dorsalis (*Extract*), 61.
Tait, L.— Ectopic Pregnancy (*Review*), 50.
Tænia, Treatment of (*Extract*), 219.
Tension, &c., Lectures on — T. Bryant (*Review*), 50.
Tetanus, Equino-telluric origin of (*Extract*), 217.

Therapeutics, Handbook of—Dr. S. Ringer (*Review*), 210.
Tibbits, Dr. H.—Massage (*Review*), 54.
Transactions of Royal Academy of Medicine in Ireland (*Review*), 211.
Tuckey, Dr. C. L.—Psycho-therapeutics (*Review*), 269.

Uterus, Displacements of—Dr. B. S. Schultze (*Review*), 52.

Vaccination—Dr. E. Warlomont, Dr. J. C. McVail, Dr. W. Woodward (*Reviews*), 44.

Warlomont, Dr. E.—Animal vaccination (*Review*), 44.
Weil's disease (*Extracts*), 60, 280.
Weston-super-Mare—C.V.Hitchins, 198.
Williams, Dr. P. W.—Chronic pharyngitis, 189.
Women, Diseases of—Dr. A. H. N. Lewers (*Review*), 51; Dr. A. J. C. Skene (*Review*), 125.
Woodward, Dr. W.—A pamphlet on vaccination (*Review*), 45.

Year-book of treatment (*Review*), 54.

### *PUBLICATIONS RECEIVED.*

From 5 Avenue de l'Opéra, Paris:
*Annales d'Orthopédie et de Chirurgie pratiques.* Nos. 18—21, 23. 1889.
From Robert Clarke & Co., Cincinnati:
*Medicine in the Middle Ages.* Extracts from *Le Moyen Age Medical* of Dr. Edmond Dupouy. Translated by T. C. Minor, M.D. 1889.
*The Evil that has been Said of Doctors: Extracts from Early Writers.* Collated from *Le Mal qu'on a dit des Medecins* of Dr. S. J. Witkoski. Translated, with Annotations, by T. C. Minor, M.D. 1889.
From 95 William Street, New York:
*The International Journal of Surgery,* September, October, 1889.
From Lea Brothers & Co., Philadelphia:
*The Medical News.* September 21st, 1889.
From Ev. E. Carreras, St. Louis:
*The Alienist and Neurologist.* October, 1889.
From Government Printing Office, Washington:
*Index-Catalogue of the Library of the Surgeon-General's Office, United States Army.* Vol. X. Q—Pfutsch. 1889.
From John Lopes, Singapore:
*The Singapore Free Press.* September 11th, 12th, 1889.
From Wilson & Jones, Birkenhead:
*Birkenhead Health Report.* By Francis Vacher. 1889.
From Oliver & Boyd, Edinburgh:
*Uterine Tumours Treated by Electricity.* By Thomas Keith, M.D., and Skene Keith. 1889.
From Young J. Pentland, Edinburgh:
*Studies in Clinical Medicine.* By Byrom Bramwell, M.D. Vol I. Nos. 8—12. 1889.
*Essentials of Medical Anatomy.* By H. R. Kenwood, M.B., C.M. 1889.
*Handbook of Obstetric Nursing.* By Francis W. N. Haultain, M.D., and J. Haig Ferguson, M.B. 1889.
*Pleurisy and Empyema.* By Byrom Bramwell, M.D. 1889.
*The Geographical Distribution of some Tropical Diseases.* By R. W. Felkin, M.D. 1889.
*Suppuration and Septic Diseases.* By W. Watson Cheyne, M.B. 1889.
From The Provincial Medical Journal, Leicester:
*The Operation of Excision of the Knee-joint.* By William Thomson. Reprint.
From Burroughs, Wellcome & Co., London:
*The A B C Medical Diary & Visiting List.* 1890.
From John Bale and Sons, London:
*Pasteur Institute.* Mansion House Fund. 1889.
From J. & A. Churchill, London:
*The Human Foot.* By Thomas S. Ellis. 1889.
*The Journal of the British Dental Association.* September, 1889.
*Electric Illumination of the Bladder and Urethra.* By E. Hurry Fenwick. Second Edition. 1889.
*The General Theory of Cancer-formation.* By Herbert Snow, M.D. 1889.
*Insane Poor in Yorkshire.* By D. Hack Tuke, M.D. 1889.
*Massage in der Frauenheilkunde.* Von Dr. L. Prochownick. 1890. (Leopold Voss, Hamburg.)
*Hospital Practice and Physical Diagnosis.* By Christopher J. Nixon, M.D. 1889.
*On the Knee-joint.* By Herbert Wm. Allingham. 1889.
From Charles Griffin and Company, London.
*A Contribution to the Surgery of the Spinal Cord.* By William Thorburn. 1889.
*A Treatise on Gout.* By Sir Dyce Duckworth. 1889.
From H. K. Lewis, London:
*The British Journal of Dermatology.* October, November, 1889.
*The Morbid Anatomy, Pathology, and Treatment of Hernia.* By Charles Barrett Lockwood. 1889.
*Animal Alkaloids.* By Sir William Aitken, M.D. Second Edition. 1889.
*The Town Dweller.* By J. Milner Fothergill, M.D. 1889.
*Chronic Bronchitis.* By William Murrell, M.D. 1889.
From Truslove and Shirley, London:
*Introduction to the Treatment of Disease by Galvanism.* By Skene Keith, M.B. n.d.

Foolscap 4to, 392 pp. Price Fifteen Shillings.

---

### Evenings with Shakspere.

**A HANDBOOK TO THE STUDY OF HIS WORKS,**

**WITH SUGGESTIONS FOR THE CONSIDERATION OF OTHER ELIZABETHAN LITERATURE,**

AND CONTAINING

**SPECIAL HELP FOR SHAKSPERE SOCIETIES.**

BY

**L. M. Griffiths,**

*Honorary Secretary of the "Clifton Shakspere Society."*

---

THIS work puts forth a plan for a systematic study of Shakspere and his fellow-dramatists, and has its information so arranged that it may be useful either to the individual student or to those who prefer to combine for the study of the Elizabethan Drama. It has much by which Shakspere Societies will be enabled to considerably simplify the details of their arrangements.

---

*Bristol:* J. W. ARROWSMITH, 11 QUAY STREET.

*London:* SIMPKIN, MARSHALL, HAMILTON, KENT, AND COMPANY LIMITED

*A New Volume commences with the January issue.*

THE

### INTERNATIONAL JOURNAL

OF THE

### MEDICAL SCIENCES.

EDITED BY

I. MINIS HAYS, M.D., PHILADELPHIA,

AND

SIDNEY COUPLAND, M.D., F.R.C.P., LONDON.

THE INTERNATIONAL JOURNAL OF THE MEDICAL SCIENCES is published simultaneously in Philadelphia, London, and Edinburgh every *Month.*

No Journal in any department of Science has hitherto been honoured with so large a list of eminent collaborators.

The character of the work may be judged by the past literary history of the Journal, which for two generations has been widely and honourably known as the American Journal of the Medical Sciences. Such a record, though legitimately a subject for pride, is here referred to as a pledge that THE JOURNAL, though crowned with honourable years and achievements, entered upon its new career in 1888 (as a Monthly instead of a Quarterly Journal), with mature experience and the vigour of youth.

THE JOURNAL is conducted on the same lines as formerly, and the contents are contributed partly by American Writers, and partly by Writers in Great Britain.

In its Monthly form THE JOURNAL has become more than ever the favourite medium for presenting Articles requiring some elaboration. Its main features are—

1. Original Articles and Monographs, illustrations being freely used to elucidate the text.
2. Reviews of the chief contributions to Medical Literature.
3. Monthly Summaries of the progress made in all Departments of Medicine—general as well as special.

The aim of THE JOURNAL is to give the most recent information in all departments of Medicine and Surgery, and to supply the General Practitioner, as well as the Consultant, Specialist, and Hospital Teacher, with a complete and well-digested *resumé* of current Medical Science. The essential feature of the Journal is its practical character; articles having a direct bearing on the diagnosis or treatment of disease, or on practical points connected with any department of Medicine, Surgery, or Midwifery, are consequently preferred to papers which are entirely theoretical and scientific.

Single numbers One Shilling and Sixpence each. By Post One Shilling and Ninepence each. Subscription (payable in advance) Eighteen Shillings per annum, *Post free.*

---

YOUNG J. PENTLAND,

EDINBURGH: 11 TEVIOT PLACE.

LONDON: 38 WEST SMITHFIELD, E.C.

PROSPECTUS.

### Studies in Clinical Medicine.

UNDER the above Title is published *every alternate Friday*, (Summer Session 1889 and Winter Session 1889-90), a record of some of the work done at Dr. BYROM BRAMWELL'S Out-Door Clinic in the Edinburgh Royal Infirmary.

The "STUDIES" consist of Cases drawn from all departments of Clinical Medicine. The questions put to the patient, the patient's answers, and Dr. BYROM BRAMWELL'S remarks, are, so far as circumstances allow, published *verbatim*.

Each Number comprises 16 pages crown quarto, handsomely printed on a specially made toned paper. Illustrations, consisting of Wood Engravings, Lithographs, and Chromo-Lithographs, are liberally introduced into the text.

### TERMS OF SUBSCRIPTION.

**Single Numbers 6d. each; Post free 7d.**

*Subscription for the Year (in advance), 20 Numbers, Post free—*

| | |
|---|---|
| Great Britain, . . . . | £0 10 6 |
| Countries in Postal Union, . . | 0 12 0 |
| India and the Colonies, . . . | 0 15 0 |

YOUNG J. PENTLAND,

EDINBURGH: 11 TEVIOT PLACE.

(*Opposite the Medical School.*)

LONDON: 38 WEST SMITHFIELD, E.C.

(*Adjoining St. Bartholomew's Hospital.*)

### ORDER FORM.

Mr. YOUNG J. PENTLAND,
*MEDICAL PUBLISHER*, EDINBURGH.
And at 38 WEST SMITHFIELD, LONDON, E.C.

*Please enter my name as a Subscriber to* **The International Journal of the Medical Sciences** *for 1890, for which I enclose you the sum of* EIGHTEEN SHILLINGS.

*Name*, ....................................................................................

*Address*, ................................................................................

*Date*, ......................................................................................

All Subscriptions payable in advance.

---

### ORDER FORM.

Mr. YOUNG J. PENTLAND,
*MEDICAL PUBLISHER*, EDINBURGH.
And at 38 WEST SMITHFIELD, LONDON, E.C.

*Please enter my name as a Subscriber to* **Studies in Clinical Medicine,** *by* DR. BYROM BRAMWELL, *for 1889-90, for which I enclose you the sum of* TEN SHILLINGS AND SIXPENCE.

*Name*, ....................................................................................

*Address*, ................................................................................

*Date*, ......................................................................................

All Subscriptions payable in advance.

*Cases for binding, price 7d. each, may be had of the Publisher,*
J. W. ARROWSMITH, *Quay Street, Bristol.*

